# A case of bloodstream infection caused by *Ruminococcus gnavus* without gastrointestinal involvement

**DOI:** 10.1016/j.heliyon.2023.e16011

**Published:** 2023-05-02

**Authors:** Tomoki Furutani, Hiroyuki Kitano, Kenichiro Ikeda, Satoshi Shirane, Yumiko Koba, Seiya Kashiyama, Hiroki Kitagawa, Kohei kobatake, Keisuke Hieda, Hiroki Ohge, Nobuyuki Hinata

**Affiliations:** a Department of Urology, Graduate School of Biomedical and Health Sciences, Hiroshima University, Hiroshima City, Japan; b Department of Clinical Practice and Support, Hiroshima University Hospital, Hiroshima City, Japan; c Department of Infectious Diseases, Hiroshima University Hospital, Hiroshima City, Japan

**Keywords:** *Ruminococcus gnavus*, Bloodstream infection, Piperacillin/tazobactam, Gastrointestinal involvement

## Abstract

We report a case of bloodstream infection due to *Ruminococcus gnavus* (*R. gnavus*) associated with pelvic abscess in a 74-year-old female patient undergoing radiotherapy for cervical cancer. Gram staining of positive anaerobic blood cultures revealed short chains of gram-positive cocci. Matrix-assisted laser desorption ionization time-of-flight mass spectrometry was performed directly on the blood culture bottle, and 16S rRNA sequencing identified the bacterium as *R. gnavus*. There was no leakage from the sigmoid colon to rectum on enterography, and *R. gnavus* was not found in the culture of her pelvic abscess. After the administration of piperacillin/tazobactam, her condition markedly improved. This patient with *R. gnavus* infection demonstrated no gastrointestinal involvement, whereas past published cases reported diverticulitis or intestinal damage. It is possible that bacterial translocation of *R. gnavus* occurred from the gut microbiota, due to damage to the intestinal tract caused by radiation.

## Highlights

• *Ruminococcus gnavus* infection occurred without gastrointestinal involvement.
• *R. gnavus* was identified by Gram staining, MALDI-TOF, and 16S rRNA sequencing.
• *R. gnavus* was the sole causative agent for the bacteremia.
• Treatment was switched from ampicillin/sulbactam to piperacillin/tazobactam.

## 1 Introduction

*Ruminococcus gnavus* is an anaerobic, non-spore-forming, gram-positive diplococcus that is part of the normal human intestinal flora [1]. *R. gnavus* is frequently found in human gut microbial communities and is present in more than 90% of all individuals [2]. Dysbiosis of the gut microbiota, with elevated *R. gnavus* counts, is strongly associated with Crohn's disease [3]. Human bloodstream infections caused by *R. gnavus* have been reported in only eight cases [[4], [5], [6], [7], [8], [9], [10]]. Two patients had a history of diverticulitis with concurrent bacteremia (*Escherichia coli* or *Pseudomonas aeruginosa*) [8], while two others had a history of intestinal damage [5,7]. Herein, we report a case of *R. gnavus*-related bloodstream infection where *R. gnavus* was the sole causative pathogen without any intestinal perforation.

### 1.1 Case description

A 74-year-old woman visited our hospital with fever. She had been treated with radiation for cervical cancer after total hysterectomy and had developed pelvic abscess due to bladder perforation 5 years ago. A drainage tube was placed to drain the pelvic abscesses. When she visited our hospital, purulent discharge was found in her drainage tube. Complete blood count revealed leukocytosis (14.6 × 10^9^ cells/L) and anemia (hemoglobin 82 g/L); her C-reactive protein (CRP) level was elevated (3.9 × 10^4^ μg/dL), and she was hospitalized. Ampicillin/sulbactam (3 g/kg bodyweight every 8 h) was initiated on admission because *Enterococcus faecalis* was identified in a pelvic abscess culture in the past. The blood culture test results were negative.

**Fig. 1:**
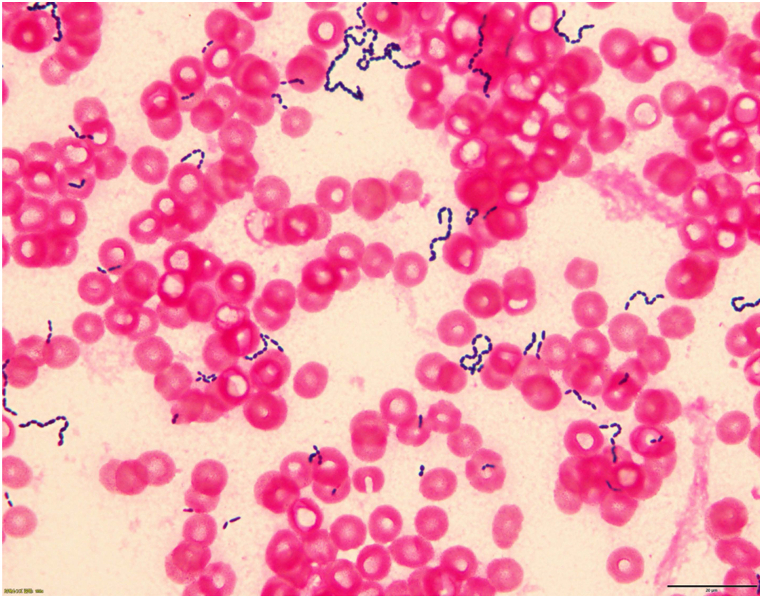
Gram staining image of *Ruminococcus gnavus* showing gram-positive diplococci arranged as short chains.

On days 2 and 3 of hospitalization, blood pressure measurement was difficult, and her pulse was 76 bpm. Computed tomography showed bleeding from the bilateral internal iliac arteries, and we performed embolization of the bilateral internal iliac arteries and stent insertion on the left external iliac artery. Her condition improved after the surgical procedure was performed without blood transfusion. On day 7 of hospitalization, she developed fever (39.1 °C). Her complete blood count revealed leukocytosis (92.00 × 10^9^ cells/L) and elevated CRP level (18.2 × 10^4^ μg/dL). We performed a blood culture again, and after incubation for 1.9 days (46 h), the two anaerobic samples were positive, while the two aerobic samples were negative. Gram staining revealed gram-positive cocci in small chains (Fig. 1). To directly identify the microorganism, blood culture extracts were analyzed using matrix-assisted laser desorption ionization time-of-flight mass spectrometry (MALDI-TOF MS), performed using a BD MALDI Biotyper Sirius system (Becton, Dickinson and Company, Franklin lakes, NJ, USA) and MBT Compass 4.1, with the MBT Compass library: Ver.9.0.0.0 (8468MSPs) (Bruker Daltonik GmbH, Bremen, Germany) database.

MALDI-TOF MS yielded a score of 2.12 for *R. gnavus*. Positive blood culture extracts were then cultured on a solid medium at 35 °C and Brucella agar medium at 37 °C. The colonies were small and translucent. The MALDI-TOF MS analysis of these colonies identified *R. gnavus* with a score of 1.98. Subsequently, 16S rRNA gene sequencing (Data associated with this study has been deposited at figshare under the accession number. 10.6084/m9.figshare.22438999. https://doi.org/10.1016/j.jiac.2021.01.005) was performed via polymerase chain reaction using the universal primers 8UA and 1485B. The amplicons were sequenced using the primers 341A, 519B, and 907A [11], and the sequences obtained from GenBank® were compared using the BLAST program (https://blast.ncbi.nlm.nih.gov/Blast.cgi, accesion no.CP027002.1), confirming *R. gnavus* (BLAST score is 100%). *R. gnavus* was not identified in the pelvic abscess culture.

To determine the bacterial susceptibility to antibiotics, we measured the minimum inhibitory concentration (MIC) of antibiotics against the strain of *R. gnavus* isolated using Etest (bioMerieux, Marcy-l’Etoile, France) on a 1-McFarland suspension of bacteria incubated for 48 h under anaerobic conditions on Brucella agar with 5% defibrinated sheep blood 1 mg/L and vitamin K1 (Becton Dickinson and Company). The breakpoints were assessed using the European Committee on Antimicrobial Susceptibility Testing (EUCAST) clinical breakpoint system (version 8.0, 2021) for gram-positive anaerobic bacteria. This strain was susceptible to penicillin (MIC: 1.5 μg/mL), ampicillin (0.5 μg/mL), ampicillin/sulbactam (<0.5 μg/mL), imipenem (1.0 μg/mL), ceftriaxone (16 μg/mL), minocycline (<0.016 μg/mL), vancomycin (0.25 μg/mL), piperacillin/tazobactam (<2 μg/mL), and clindamycin (0.125 μg/mL) but was resistant to ciprofloxacin and levofloxacin (>32 μg/mL).

*P. aeruginosa, Enterococcus faecium, and Bacteroides fragilis* were identified by simultaneously examining the pelvic abscess culture using VITEK2 Compact (Sysmex・bioMérieux, USA). The drug was switched to tazobactam/piperacillin (TAZ/PIPC, 4.5 g/kg bodyweight every 8 h).

On day 12 after starting TAZ/PIPC administration, her temperature dropped to approximately 37 °C and her CRP decreased to the pretreatment level (5.58 × 10^4^ μg/dL). Enterography revealed no leakage from the sigmoid colon to the rectum. She has not complained of abdominal pain or other abdominal symptoms for 2 years and the bacteremia caused by *R. gnavus* has not recurred.

## 2 Discussion

Anaerobic infections occur in 0.5–20% of all positive blood cultures. Anaerobic bacteremia usually occurs secondary to an infectious process that emanates from an intra-abdominal source, the oral cavity, female genital tract, respiratory tract, or skin or soft tissue infections. Anaerobic bacteremia remains associated with significant mortality, ranging from 15% to 60% [12]. *R. gnavus* is an anerobic gram-positive diplococci with 1–3 flagella [8].

Herein, we present the ninth reported case of *R. gnavus* bacteremia. Five cases of *R. gnavus* bacteremia were associated with gastrointestinal disease, of which two cases were associated with diverticulitis [8]. The other cases were associated with fecal peritonitis secondary to small bowel herniation and perforation [10], fecal peritonitis secondary to small bowel perforation [9], and intestinal perforation [5]. One patient with *R. gnavus* bacteremia had no gastrointestinal disease, and it was considered that *R. gnavus bacteremia* was caused by bacterial translocation of the gut microbiota due to prednisolone treatment of multiple myeloma and myelodysplastic syndrome [6]. In addition to blood, *R. gnavus* has been reported to be detected in the joint fluid of a prostatic implant [13] in a patient who had been previously treated for a pelvic abscess due to the effect of radiation therapy for cervical cancer and had no history of gastrointestinal perforation or intestinal disease. However, *R. gnavus* bacteremia was caused by the bacterial translocation of *R. gnavus* from the gut microbiota, which may have been due to damage to the intestinal tract caused by radiation. Thus, infection in the present case was possibly caused by translocation of *R. gnavus* from the intestinal tract damaged due to pelvic abscesses or radiation therapy for cervical cancer.

Previous *R. gnavus* infections reported susceptibility to penicillin and amoxicillin/clavulanic acid, meropenem, imipenem, cefotaxime, tetracycline, metronidazole, clindamycin, vancomycin, and piperacillin/tazobactam [[5], [6], [7], [8],^10^]. In the present case, the infection in the patient was susceptible to penicillin, ampicillin, ampicillin/sulbactam, imipenem, ceftriaxone, minocycline, vancomycin, piperacillin/tazobactam, and clindamycin. We obtained favorable results with TAZ/PIPC treatment. However, other antimicrobials are also sensitive to *R. gnavus*; Since there are few reports of Ruminococcus infections, it is considered necessary to accumulate a large number of cases in order to select the optimal antimicrobial drug. Since Ruminococcus is an intestinal bacterium, one must look for gastrointestinal lesions if one develops bacteremia with the organism. If there is no such cause, it is necessary to further search for causes such as the effects of radiation, immunosuppressive drugs, or immune disorders, and then bacterial translocation from the gastrointestinal tract should be suspected and treated.

### 2.1 Patient consent

Written informed consent was provided by the patient's legal guardian for the publication of this case report.

## Author contribution statement

All authors listed have significantly contributed to the investigation, development and writing of this article.

## Data availability statement

Data associated with this study has been deposited at figshare under the accession number. 10.6084/m9.figshare.22438999 (https://doi.org/10.1016/j.jiac.2021.01.005).

## Declaration of competing interest

The authors declare that they have no known competing financial interests or personal relationships that could have appeared to influence the work reported in this paper.

## References

[1] C Moore W.E., Johnson J.L., Holdeman L.V. (1976). Emendation of *Bacteroidaceae* and *Butyrivibrio* and descriptions of *Desulfomonas* gen. nov. and ten new species in the genera *Desulfomonas, Butyrivibrio, Eubacterium, Clostridium*, and *Ruminococcus*. Int. J. Syst. Evol. Microbiol..

[2] Qin J., Li R., Raes J., Arumugam M., Burgdorf K.S., Manichanh C. (2010). A human gut microbial gene catalogue was established by metagenomic sequencing. Nature.

[3] Joossens M., Huys G., Cnockaert M., De Preter V., Verbeke K., Rutgeerts P. (2011). Dysbiosis of the faecal microbiota in patients with Crohn's disease and their unaffected relatives. Gut.

[4] Fan X., Chen Y., Liu Y., Hu L. (2022). First case of bloodstream infection caused by *Ruminococcus gnavus* in an 85 year old man in China. Lab. Med..

[5] Fontanals D., Larruzea A., Sanfeliu I. (2018). Direct identification of *Ruminococcus gnavus* by matrix-assisted laser desorption/ionization time-of-flight mass spectrometry (MALDI-TOF MS) in a positive anaerobic blood culture bottle. Anaerobe.

[6] Gren C., Spiegelhauer M.R., Rotbain E.C., Ehmsen B.K., Kampmann P., Andersen L.P. (2019). *Ruminococcus gnavus* bacteremia in a patient with multiple haematological malignancies. Access Microbiol.

[7] Kim Y.J., Kang H.Y., Han Y., Lee M.S., Lee H.J. (2017). A bloodstream infection by *Ruminococcus gnavus* in a patient with gallbladder perforation. Anaerobe.

[8] Hansen S.G., Skov M.N., Justesen U.S. (2013). Two cases of *Ruminococcus gnavus* bacteremia were associated with diverticulitis. J. Clin. Microbiol..

[9] Struyve M., De Vloo C., Lefever S., Boudewijns M., De Bel A., D'Hondt M. (2018). *Ruminococcus gnavus* bacteremia associated with fecal peritonitis secondary to small bowel perforation. Acta Gastroenterol. Belg..

[10] Lefever S., Van Den Bossche D., Van Moerkercke W., D'Hondt M., Alegret Pampols M.D.C., Struyve M. (2018). *Ruminococcus gnavus* bacteremia is an uncommon presentation of a common member of the human gut microbiota: a case report and literature review. Acta Clin. Belg..

[11] Imoto W., Takahashi Y., Yamada K., Hojo K., Kawase T., Sakon Y. (2021). *Corynebacterium jeikeium*-induced infective endocarditis and perivalvular abscess diagnosed by 16S ribosomal RNA sequence analysis: a case report. J. Infect. Chemother..

[12] De Keukeleire S., Wybo I., Naessens A., Echahidi F., Van der Beken M., Vandoorslaer K. (2016). Anaerobic bacteraemia: a 10-year retrospective epidemiological survey. Anaerobe.

[13] Simmon K.E., Mirrett S., Reller L.B., Petti C.A. (2008). Genotypic diversity of anaerobic isolates from bloodstream infections. J. Clin. Microbiol..

