# Supplementary material for: A case of bloodstream infection caused by Ruminococcus gnavus without gastrointestinal involvement
Source: Heliyon. 2023 May 2;9(5):e16011. doi: 10.1016/j.heliyon.2023.e16011 (PMC10192738; doi:10.1016/j.heliyon.2023.e16011)
Supplement: Multimedia component 1 [file mmc1.docx]

The sequence of the primers

341A

CGACGCCGCGTGAGCGATGAAGTATTTCGGTATGTAAAGCTCTATCAGCAGGGAAGAAAATGACGGTACCTGACTAAGAAGCCCCGGCTAACTACGTGCCAGCAGCCGCGGTAATACGTAGGGGGCAAGCGTTATCCGGATTTACTGGGTGTAAAGGGAGCGTAGACGGCATGGCAAGCCAGATGTGAAAGCCCGGGGCTCAACCCCGGGACTGCATTTGGAACTGTCAGGCTAGAGTGTCGGAGAGGAAAGCGGAATTCCTAGTGTAGCGGTGAAATGCGTAGATATTAGGAGGAACACCAGTGGCGAAGGCGGCTTTCTGGACGATGACTGACGTTGAGGCTCGAAAGCGTGGGGAGCAAACAGGATTAGATACCCTGGTAGTCCACGCCGTAAACGATGAATACTAGGTGTCGGGTGGCAAAGCCATTCGGTGCCGCAGCAAACGCAATAAGTATTCCACCTGGGGAGTACGTTCGCAAGAATGAAACTCAAAGGAATTGACGGGGACCCGCACAAGCGGTGGAGCATGTGGTTTAATTCGAAGCAACGCGAAGAACCTTACCTGGTCTTGACATCCCTCTGACCGCTCTTTAATCGGAGCTTTCCTTCGGGACAGAGGAGACAGGTGGTGCATGGTTGTCGTCAGCTCGTGTCGTGAGATGTTGGGTTAAGTCCCG

907A

CCTTACCTGGTCTTGACATCCCTCTGACCGCTCTTTAATCGGAGCTTTCCTTCGGGACAGAGGAGACAGGTGGTGCATGGTTGTCGTCAGCTCGTGTCGTGAGATGTTGGGTTAAGTCCCGCAACGAGCGCAACCCCTATCTTTAGTAGCCAGCATTTTGGATGGGCACTCTAGAGAGACTGCCAGGGATAACCTGGAGGAAGGTGGGGATGACGTCAAATCATCATGCCCCTTATGACCAGGGCTACACACGTGCTACAATGGCGTAAACAAAGGGAAGCGAGCCCGCGAGGGGGAGCAAATCCCAAAAATAACGTCTCAGTTCGGATTGTAGTCTGCAACTCGACTACATGAAGCTGGAATCGCTAGTAATCGCGAATCAGAATGTCGCGGTGAATACGTTCCCGGGTCTTGTACACACCGCCCGTCACACCATGGGAGTCAGTAACGCCCGAAGTCAGTGACCCAACCGCAAGGAGGGAGCTGCCGAAGGTGGGACCGATAACTGGGGTGAAGTCGT

519B

CGTCATTTTCTTCCCTGCTGATAGAGCTTTACATACCGAAATACTTCATCGCTCACGCGGCGTCGCTGCATCAGGGTTTCCCCCATTGTGCAATATTCCCCACTGCTGCCTCCCGTAGGAGTTTGGGCCGTGTCTCAGTCCCAATGTGGCCGGTCACCCTCTCAGGTCGGCTACTGATCGTCGGCTTGGTAGGCCGTTACCCCACCAACTACCTAATCAGACGCGGGTCCATCTCATACCACCGGAGTTTTTCACACCGTACCATGCGGTACTGTGCGCTTATGCGGTATTAGCAGCCGTTTCCAACTGTTATCCCCCTGTATGAGGCAGGTTACCCACGCGTTACTCACCCGTCCGCCGCTCAGTCACCAAGGCTTCAATCCGAAGAAATCCGTCAAGGTGCTTCGCTCGACTTGCATGTGTTAAGCACGCCGCCAGCGTTCATCCT
